# Intermittent Fasting May Enhance Resistance Training Effects on the Body Composition of Obese Males, Without Affecting Muscular Strength and Anabolic Index

**DOI:** 10.1155/jobe/6409069

**Published:** 2026-02-19

**Authors:** Ali Farahmand Khoshkebijari, Maryam Ebrahimi, Abuzar Jorbonian

**Affiliations:** ^1^ Department of Exercise Physiology, Faculty of Physical Education and Sport Sciences, University of Guilan, Rasht, Guilan, Iran, guilan.ac.ir

**Keywords:** cortisol, fat mass, muscular strength, testosterone

## Abstract

**Purpose:**

This randomized controlled trial aimed to evaluate the impact of intermittent fasting (IF) during resistance training (RT) on body composition, muscular strength, and the testosterone:cortisol ratio in obese males.

**Methods:**

Twenty obese males (aged 20–30, BMI 30–36 kg/m^2^) were selected from eligible volunteers and randomly assigned to control (regular diet) and IF (4:3 IF) groups. All subjects participated in RT 3 days/week for 8 weeks. Forty‐eight hours before and after the protocol, blood sampling and anthropometric measurements were done in a fasting state, and data were analyzed at a significance level of *p* < 0.05.

**Results:**

The IF/RT group lost twofold more weight and fat, had higher arm and chest measurements, and had less waist circumference than the C/RT group. The testosterone levels and muscle strength improved with RT, and there was no difference between the C/RT and IF/RT groups.

**Conclusion:**

It appears that intermittent fasting may enhance the efficacy of RT in obese males and is unlikely to have any detrimental effects on muscular strength or the anabolic index.

**Trial Registration:** Iranian Registry of Clinical Trials (IRCT): IRCT20190213042702N4

## 1. Introduction

The rising worldwide prevalence of obesity, also known as “globesity,” poses a significant and pressing public health concern for communities and healthcare infrastructures. The global impact of a high body mass index (BMI) affects every country, with low‐income nations experiencing the greatest increases over the past decade. According to the World Obesity Atlas 2024 report, 79% of adults with overweight and obesity will live in low‐ and middle‐income countries by 2035 [1].

The significant surge in obesity rates has been the subject of extensive research. Potential contributing factors include increased caloric intake, changes in diet, decreased physical activity levels, and alterations in the gut microbiome. Various kinds of evidence indicate that the primary driving force behind the obesity epidemic is increased energy intake, while reduced physical activity plays a lesser role [2]. Studies have reported that a 5% weight loss produces clinically significant improvements in obesity‐associated conditions [3].

Intermittent fasting (IF) is one of the strategies for weight management and is characterized by alternating periods of little or no caloric intake and normal food consumption. IF may involve consuming daily caloric intake within a specific time window, such as 8 hours, fasting one day, and consuming food the next day. On nonfasting days, food consumption may be unrestricted, limited to a specific diet, or aimed at achieving a caloric intake of up to 125% of regular caloric needs [4].

There are several types of IF. Time‐restricted feeding (TRF) involves limiting daily food intake to a specific time window, typically lasting no more than 10 h. Periodic fasting (PF) entails fasting one to 2 days per week, with unrestricted food consumption for the remaining five to 6 days. Alternate‐day fasting (ADF) involves fasting 24 h on alternate days [5]. The weight loss mechanism by IF is associated with factors such as consuming fewer calories, increased fat burning, improved insulin sensitivity, and better glucose metabolism [6]. When fasting, triglycerides are broken down into fatty acids and glycerol, which the liver converts into ketone bodies. This process provides a significant energy source for many tissues and helps lose fat, which could lead to positive changes in body composition [7]. IF also facilitates a mechanism wherein nutrients are recycled during food deprivation, sustaining cellular energy equilibrium and enhancing the immune system [8].

IF is known to influence the body’s internal circadian clocks, which regulate the release of metabolites and hormones such as cortisol, testosterone, insulin, and glucagon [9]. The testosterone:cortisol ratio was previously used to indicate anabolic balance [10]. IF, particularly in the form of time‐restricted eating, has been associated with significant testosterone declines. Despite this decline, studies consistently reported no impairment in muscular development or performance [11]. Additionally, cortisol levels appear to be sensitive to variables such as nutrient intake, glycemic status, and age. IF causes hyperstimulation of the hypothalamic pituitary adrenal axis, resulting in mild cortisol elevations. The effects of fasting on the testosterone:cortisol ratio have undergone little investigation [12]. However, investigations have found no significant variation in the testosterone:cortisol ratio following 24 h of fasting [13]. Reducing energy intake is a primary approach to reducing body fat [14]. But considering the effects of fasting on anabolic–catabolic pathways, one of the potential adverse effects of limiting energy intake is the loss of lean body mass (LBM). The preservation of skeletal muscle mass (SMM) is influenced by muscle protein synthesis (MPS) rates and muscle protein breakdown (MPB). When subject to energy restriction or fasting, there is a possibility that the rates of MPB could accelerate while the rates of MPS decrease. Consequently, this imbalance can reduce net protein balance and may, to some extent, contribute to the decline in LBM [15]. LBM plays a major role in basal metabolic rate (BMR), so losing LBM can decrease energy expenditure and make it harder to lose weight. Reduced LBM can affect metabolic health and increase the risk of disease in overweight and obese individuals [16]. Thus, strategies to prevent LBM loss in fasting diets should be considered. Resistance training (RT) is a powerful stimulus for increasing SMM, and it is widely recognized that combining RT with dietary approaches is an effective strategy for enhancing overall body composition [14]. Orange et al. [16] reported that RT has a large positive effect on muscle strength and a moderate effect on physical function in adults who are overweight or obese. Although concomitant calorie restriction may compromise the functional adaptations to RT, it is reported that RT performed in fasted and fed states appears to have similar effects on body composition, muscle hypertrophy, and strength of adults [17].

Based on several clinical practice guidelines and scientific societies, current research on IF is limited. Therefore, it is recommended that a lifestyle intervention that integrates dietary and physical activity components be provided as a fundamental aspect of any weight management intervention. Therefore, this research aims to assess the combined effects of IF with RT on changes in body composition and testosterone/cortisol ratio in sedentary, obese males.

## 2. Methods

### 2.1. Study Design

This randomized controlled trial, based on CONSORT, was conducted in two control and experimental groups. It involved 2 months of IF and RT in sedentary obese males.

### 2.2. Participants

**FIGURE 1:**
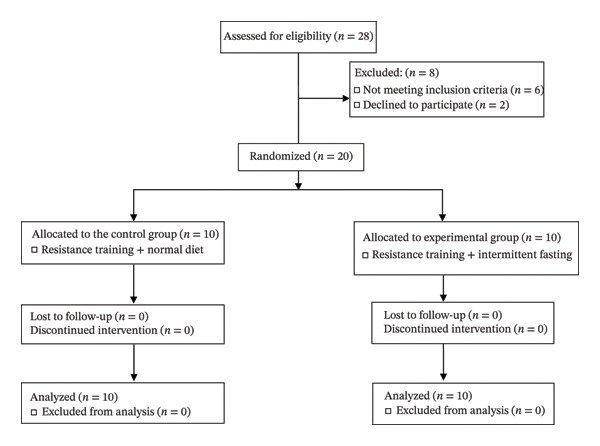
CONSORT flow diagram.

Following an announcement, 20 men (calculated by G power software at a significance level of α = 0.05, statistical power 80%, and effect size 0.5, for repeated measures ANOVA) were selected from volunteers. The inclusion criteria consisted of sedentary men aged 20–30 years, without regular resistance or aerobic training history for the last 6 months, having a BMI between 30 and 36 kg/m^2^, without a history of cardiovascular diseases or musculoskeletal injuries (Table 1). The participants were fully informed about the study protocol verbally and in writing, signed an informed consent form, and were randomly divided into C/RT (normal diet + RT; *n* = 10) and IF/RT (IF + RT; *n* = 10) groups (Figure 1). The random allocation method was used in this study so that the red (intervention) and blue (control) cards were placed inside the envelope and were randomly given to the participants by a third person.

**TABLE 1:** Participants’ characteristics (mean ± SD).

	C/RT group (*n* = 10)	IF/RT group (*n* = 10)	*p* value
Age (years)	25.50 ± 3.59	24.40 ± 2.83	0.542
Height (m)	1.77 ± 0.02	1.77 ± 0.03	0.082
Weight (kg)	107.80 ± 4.77	103.40 ± 5.85	0.540
BMI (kg/m^2^)	34.07 ± 1.62	32.95 ± 1.36	0.114

*Note:* C/RT: normal diet + resistance training; IF/RT: intermittent fasting + resistance training.

### 2.3. Procedure

Blood sampling and anthropometric measurements were done 48 h before the protocol initiation, following 12 h of overnight fasting at 08:00 a.m. Height and weight were measured using a medical SECA instrument. Participants were asked to be placed on a scale with minimum clothing and an empty bladder and gut. The weight and standing height were recorded in a similar condition, pre‐ and posttraining protocol, by a single professional. Circumferences were measured in centimeters using a soft tape measure on the right side of the body. The arm circumference was measured in a bent and contracted situation. The circumference of the chest and the midthigh was measured in a standing position [18]. Fat% was estimated by the Lafayette caliper model 12‐1110 according to the 3‐site skinfold method [19] and the Siri equation [20]. Quadriceps muscle cross‐sectional area (CSA) was calculated as QCSA (cm^2^) = (2.52 × midthigh circumference (cm)) – (1.25 × anterior thigh skinfold thickness (mm)) – 45.13 [18]. A single professional person performed all measurements in a similar situation in the morning and in a fasted condition.

One repetition maximum (1RM) was estimated for each exercise [21]. 1RM estimation for lower and upper body exercises was performed in an alternate order with enough rest. Our self‐designed protocol, based on real experiments in gyms, consisted of RT with 70% 1RM, 3 days/week for 8 weeks. At 11:00 a.m. on odd days, participants performed 7 workouts, including bench press, deadlift, barbell back squat, hamstring curl, standing dumbbell shoulder press, standing barbell curl, and standing dumbbell overhead triceps extension. The volume of exercises increased gradually using rest‐pause and drop‐set training systems:

• Weeks 1 and 2: 70% of 1RM, 10 repetitions, 3 sets
• Weeks 3 and 4: 70% of 1RM, 10 repetitions, 4 sets
• Weeks 5 and 6: 70% of 1RM, 10 repetitions, 4 sets, rest‐pause technique in the first set
• Weeks 7 and 8: 70% of 1RM, 10 repetitions, 4 sets, drop‐set technique in the last set

Training load was matched in both groups [22]. The control group continued their normal diet through the study protocol and consumed a standard meal (300 kcal) up to two hours before exercise. Fasting started 12 h before the exercise session. In each following session, 1 h was added to the fasting time to reach the desired fasting time (18 h). The training intervention was supervised by a professional trainer.

Forty‐eight hours after the last session of 8 weeks of training and after 12 h of overnight fasting, blood samples were recollected at 08:00 a.m., and anthropometric assessments were repeated. Plasma testosterone was measured using the AccuBind ELISA kit (sensitivity: 0.038 ng/mL, reading: 450 nm), and cortisol was measured using the DetectX ELISA kit (sensitivity: 27.6 pg/mL, reading: 450 nm).

### 2.4. Statistical Methods

All values are presented as mean ± standard deviation (SD). Data analysis was conducted using the Statistical Package for the Social Sciences (SPSS Version 27.0, Chicago, IL) at the significance level of *p* ≤ 0.05. Data distribution was tested by the Shapiro–Wilk test; in case of an abnormal data distribution, the inverse df method using fractional ranks was used for normalizing the data with the actual mean and SD of the distribution. A repeated measures analysis of covariance (RM ANCOVA), with pretest measures as a covariate, was used for statistical analysis. When a significant F value was achieved, *P* *η* ^2^ *and* *ω* ^2^ are reported. GraphPad Prism (Version 10.2.2) was applied to illustrate graphs.

## 3. Results

### 3.1. Body Composition

The mean weight decreased by ∼5.4% in C/RT and ∼9.8% in IF/RT groups. However, this effect disappeared when baseline weight was included as a covariate (*p* = 0.068). The difference in postintervention weight between groups was statistically significant (*F = *18.64, *p* < 0.001, *P* *η* ^2^ *= *0.523, *ω* ^2^ *= *0.48), and the IF group showed a lower weight (Figure 2(a)).

FIGURE 2Mean ± SD of (a) weight and (b) fat% in groups. The IF group showed lower weight and fat% compared with the control group. ^#^Significant difference between groups at *p* < 0.05.

**Figure:**
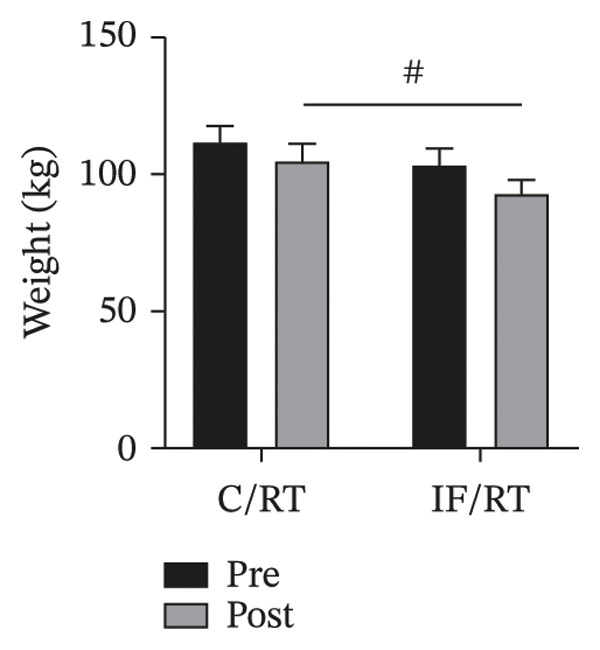
(a)

**Figure:**
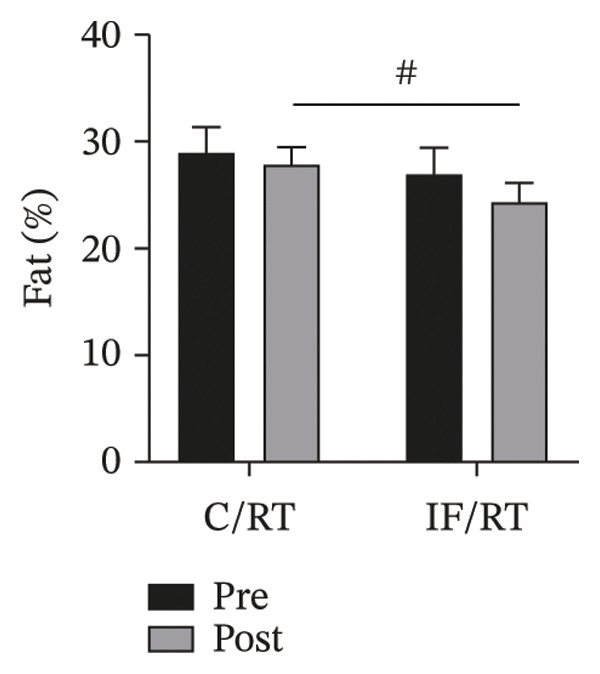
(b)

The fat percentage decreased by ∼6% in the C/RT and ∼12% in the IF/RT groups. However, this effect disappeared when baseline fat% was included as a covariate (*p* = 0.764). The difference in postintervention fat% between groups was significant (*F = *25.26, *p* = 0.001*,* *P* *η* ^2^ *= *0.598, *ω* ^2^ *= *0.561) (Figure 2(b)).

### 3.2. Circumferences

The thigh circumference decreased by ∼3% in the C/RT group and increased by ∼0.6% in the IF/RT group. However, the difference was not statistically significant (*p* = 0.869). Considering the pretest values, the mean postintervention thigh circumference was significantly higher in the IF/RT group than in the C/RT group (*F = *24.92, *p* = 0.001, *P* *η* ^2^ *= *0.594, *ω* ^2^ *= *0.557) (Figure 3(a)).

FIGURE 3Mean ± SD of (a) thigh, (b) arm, (c) chest, and (d) waist circumferences and (e) quadriceps cross‐sectional area (QCSA). The IF group experienced more hypertrophy than the control group.^∗^Significant *within-group* effect. ^#^Significant between‐group effect (*p* < 0.05).

**Figure:**
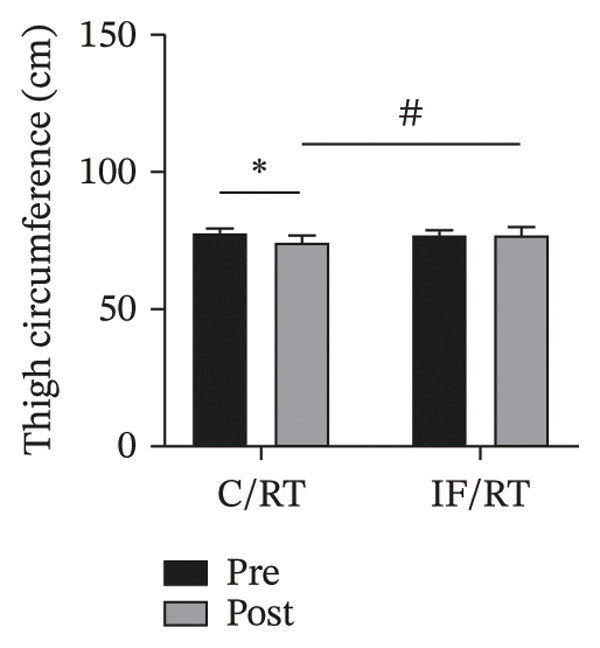
(a)

**Figure:**
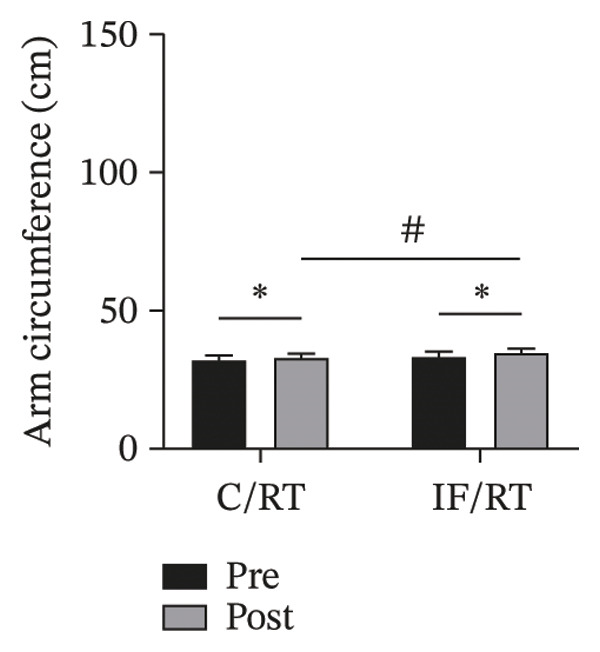
(b)

**Figure:**
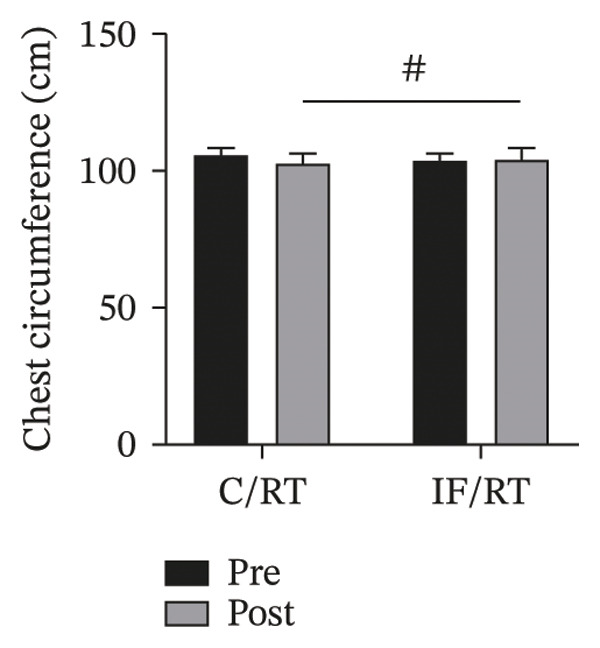
(c)

**Figure:**
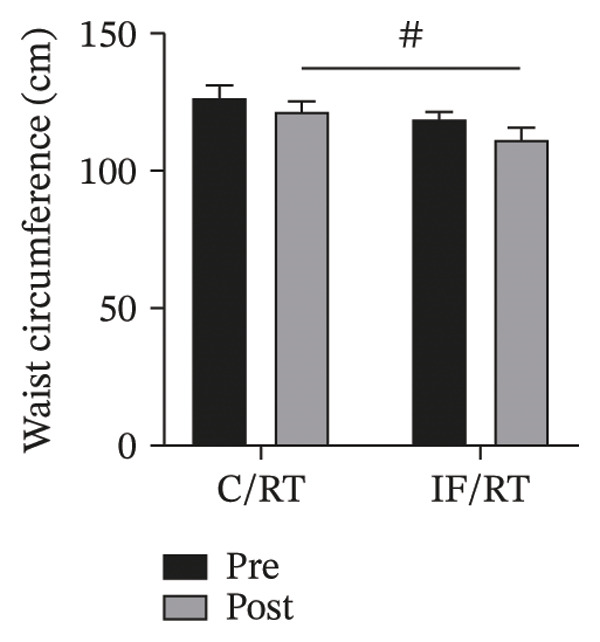
(d)

**Figure:**
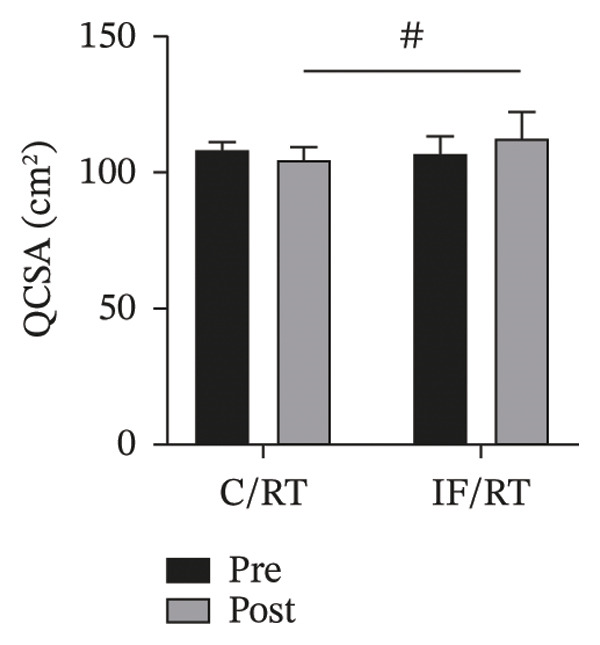
(e)

The arm circumference increased by ∼4% in the C/RT and ∼6% in the IF/RT groups (*F = *20.53, *p* < 0.001, *P* *η* ^2^ *= *0.547, *ω* ^2^ *= *0.507), and the difference in postintervention arm circumference was also significant between groups (*F = *8.20, *p* = 0.011, *P* *η* ^2^ *= *0.326, *ω* ^2^ *= *0.275) (Figure 3(b)).

While the chest circumference decreased by ∼2% in C/RT, the IF/RT group showed a ∼2% increase. Considering pretests as covariates, the differences were not statistically significant (*p* = 0.132). The difference of posttests was statistically significant between groups (*F = *23.84, *p* = 0.001, *P* *η* ^2^ *= *0.584, *ω* ^2^ *= *0.546) (Figure 3(c)).

The waist circumference was reduced by ∼3% in the C/RT and ∼6% in the IF/RT groups, but it was not statistically significant (*p* = 0.077). Postintervention value, however, was significantly lower in the IF group (*F = *9.78, *p* = 0.006, *P* *η* ^2^ *= *0.365, *ω* ^2^ *= *0.316) (Figure 3(d)).

Although the QCSA showed a ∼3% decrease in C/RT and ∼6% increase in the IF/RT groups, it was not significant (*p* = 0.115). However, the difference between posttest values was significant between groups (*F = *37.35, *p* < 0.001, *P* *η* ^2^ *= *0.687, *ω* ^2^ *= *0.656) (Figure 3(e)).

### 3.3. Muscular Strength

Leg press 1RM increased ∼40% in C/RT (*p* = 0.001) and ∼46% in IF/RT (*p* = 0.012), but between groups, the effect was not statistically significant (*p* = 0.852) (Figure 4(a)). Bench press 1RM increased by ∼46% in C/RT (*p* = 0.001) and ∼36% in IF/RT (*p* = 0.001), but the between‐group effect was not statistically significant (*p* = 0.132) (Figure 4(b)).

FIGURE 4Mean ± SD of (a) leg and (b) bench press in groups. ^∗^Significant within‐group effect. Resistance training improved upper and lower body strength and IF had no extra effect on it.

**Figure:**
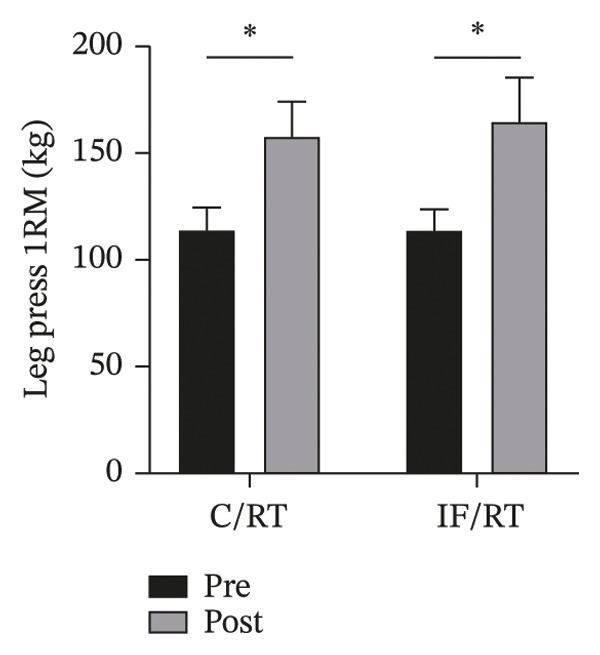
(a)

**Figure:**
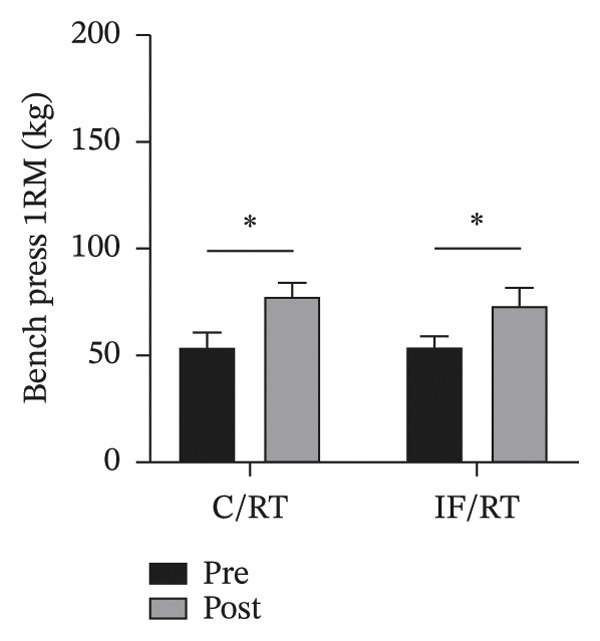
(b)

### 3.4. Cortisol/Testosterone Levels

Cortisol levels decreased after 8 weeks of RT in the C/RT and IF/RT groups, but the difference based on pretests as a covariate was not statistically significant in groups (*p* = 0.469) or between groups (*p* = 0.540) (Figure 5(a)).

FIGURE 5Mean ± SD of (a) cortisol, (b) testosterone, and (c) testosterone:cortisol ratio in groups. Resistance training improved only testosterone levels, and IF did not affect the anabolic/catabolic state. ^∗^Significant within‐group effect.

**Figure:**
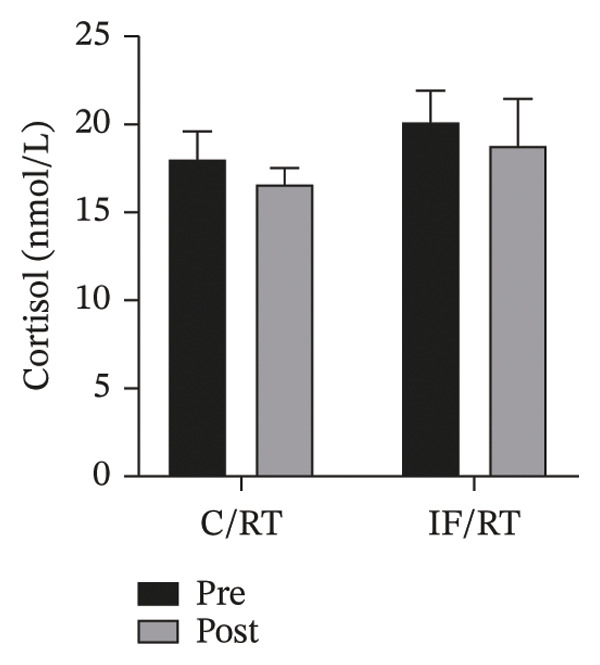
(a)

**Figure:**
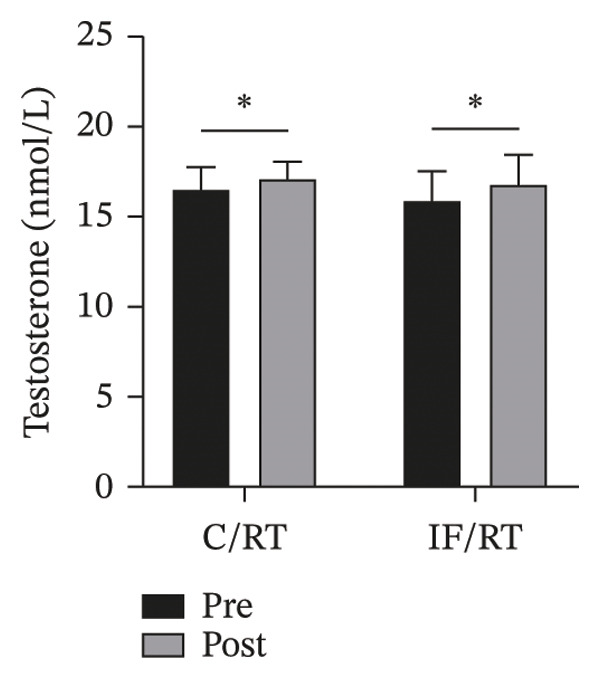
(b)

**Figure:**
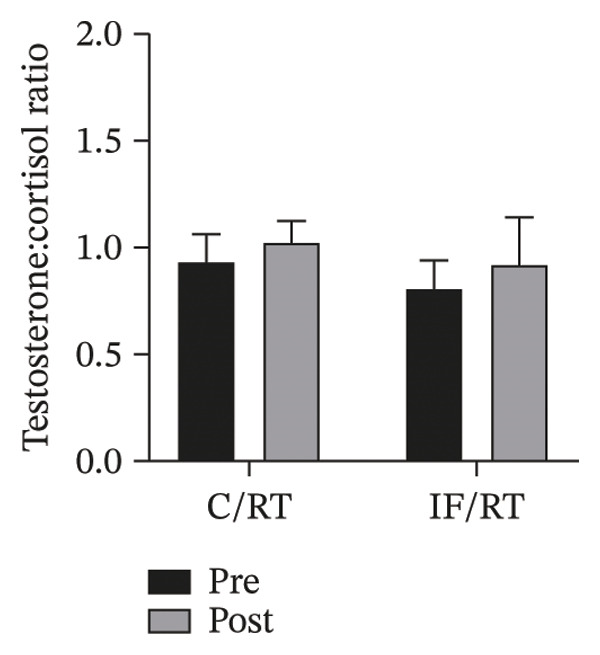
(c)

Testosterone levels were significantly increased after 8 weeks in the C/RT and IF/RT groups (*F = *7.86, *p* = 0.012*,* *P* *η* ^2^ *= *0.316, *ω* ^2^ *= *0.265), but the posttest levels between groups were not significantly different (*p* = 0.581) (Figure 5(b)).

The testosterone:cortisol ratio increased in C/RT and IF/RT. However, considering the pretest levels as covariate, the difference was not significant (*p* = 0.409). Also, the difference of posttests between groups was not significant (*p* = 0.861) (Figure 5(c)).

## 4. Discussion

IF diets have become very popular recently, as they can safely produce clinically significant weight loss [23]. The IF strategies have been applied in short durations, and the effect of IF concomitant with exercise is still unclear. For this reason, we investigated the effect of eight weeks of RT with 70% 1RM with either an 18‐hour IF or a regular diet in obese males.

Almost all IF studies have resulted in some degree of weight loss, ranging from 2.5% to 9.9% [8]. We also observed a ∼ 9.8% decrease in body weight through 8 weeks of RT with IF, which was nearly twofold more than the weight loss in RT with a regular diet. However, when baseline values were included as a covariate, this effect was diminished, and it seems that lower postintervention weight in the IF group is more related to the fasting itself. It has been reported that ADF plus exercise could decrease fat mass and favorably affect plasma lipids, while each alone had no significant effect [24]. In the present study, the fat% and waist circumference reduction in IF/RT were twofold higher than those in the C/RT groups. Considering baseline levels, there was no significant effect of training. Therefore, the reduced weight after 8 weeks could be related to the IF‐induced fat mass reduction. Most studies examining fasts of < 48 h demonstrate an increase of approximately 5.5% in energy expenditure within the first 24 h of fasting, with the majority occurring between 18 and 24 h. Many of the alterations displayed within the early stages of fasting are believed to occur in response to changes in circulating catecholamines and sympathetic nervous system (SNS) activity [10]. However, the rise in epinephrine was only seen in nonendurance‐trained subjects, suggesting exercise training status may also be a noteworthy factor influencing catecholamine responses to acute fasting [25]. The contributions of individual catecholamines are yet to be fully elucidated.

Interestingly, chest and thigh circumference and QCSA decreased in the C/RT group, while the arm and chest circumference and QCSA in the IF/RT group increased. It seems that training had more effect on arm circumference, while fasting had more effect on thigh hypertrophy. Moro et al. [26] reported no significant changes in LBM over the 8 weeks following TRF with an 8‐hour feeding window or control diet. However, the TRF group significantly reduced their fat mass. Stratton et al. [27] also reported that participants with TRF with an 8‐h feeding window compared to a control diet lost significant body weight, fat mass, and body fat percentage but experienced no change in LBM. In one study that investigated modified IF [28], the authors combined ADF with concurrent exercise in obese individuals. Findings revealed a significant decrease in LBM in the modified ADF plus exercise group from baseline, though this was not statistically different from the customary diet–only group. However, a systematic review concluded that LBM is generally maintained when IF is combined with RT. However, whether or not IF inhibits LBM accrual is unclear and may be contingent on the adequate provision of protein and energy balance [29].

Muscle strength improved after 8 weeks of RT, and IF did not affect the participants’ 1RM. According to a meta‐analysis, 7 studies have also reported no significant changes in muscle strength when comparing pre‐ and post‐IF time points [30]. Kerr [31] suggested that IF decreases the muscles’ ability to perform at the same level as before the fast, but other studies show that it does not harm sports performance and could be considered a suitable diet for sports practice [32].

Two crucial endocrine hormones greatly affected by obesity are testosterone and cortisol. Testosterone is an anabolic hormone that may be important for muscle strength and hypertrophy. It has been shown that obese males experience lower testosterone and higher cortisol levels, which may affect anabolic/catabolic pathways [33]. We observed improved testosterone levels after RT, while IF did not affect this hormone. Also, cortisol and testosterone‐to‐cortisol ratio were not affected by training or IF. Research has shown that RT elevates testosterone levels in lean individuals [34]. The testosterone generated in response to resistance exercise significantly preserves LBM by increasing MPS and causing muscle hypertrophy [35]. In addition to elevating BMR and LBM, RT provides other benefits that improve glycemic control and help prevent diabetes and metabolic syndrome [36]. It has also been reported that the hypertrophy training protocol elicits a greater testosterone response compared to other protocols [37]. Other studies in the literature also support the idea that a decrease in body fat percentage reduces the testosterone response to resistance exercise [38].

Testosterone is reported to be reduced with IF [26]. Meanwhile, a meta‐analysis of studies on the obese failed to show changes in cortisol with IF diets, with the authors citing large bias for all parameters studied [39]. Al‐Rawi et al. [40] also reported that diurnal IF after 1 month did not affect the cortisol levels of obese adults. Theoretically, one would expect fasting to result in an elevation of cortisol levels for energy mobilization. The circadian rhythm plays a significant role in regulating cortisol levels. Alterations in dietary patterns due to changes in meal timing can impact the daily cortisol profile. In individuals with obesity, a study has reported an enhancement in cortisol rhythmicity after following an IF schedule involving a 10‐hour fast starting from 7:30 p.m. for eight weeks. This reduced the morning cortisol area under the curve, while the evening cortisol levels remained unaltered [41]. The act of skipping breakfast or dinner yields distinct physiological outcomes. Skipping dinner reduces evening cortisol levels, resulting in a statistically insignificant rise in morning cortisol. Conversely, omitting breakfast leads to a notable reduction in morning cortisol levels [42]. However, in our 8‐week protocol, morning cortisol levels did not change with RT or IF.

Our research had some limitations, including the relatively small sample size and lack of individual control of diet in the feeding window. Considering relatively large effect sizes, our data could be reliable despite its small size. It was difficult to match dietary intake during the 8 weeks of the program. Although a specific diet would be another independent factor that should be considered in another investigation, future studies may consider food recalls. Sleep quantity or quality during the program may also affect results, which we did not control. However, we asked participants to continue their regular lifestyle during the protocol.

## 5. Conclusion

This study showed that RT with IF may improve fat loss and weight control in obese males, with no negative effect on the anabolic index and muscular strength. The physiological effect of IF on obesity treatment needs more investigation, and we suggest that the time point and duration of fasting, dietary intake, and sleep be considered in future studies.

## Author Contributions

Ali Farahmand Khoshkebijari carried out the literature study and executed the protocol. Maryam Ebrahimi participated in the study design, statistical analysis, and manuscript writing. Abuzar Jorbonian participated in the study design and data interpretation and helped draft the manuscript.

## Funding

This research did not receive specific funding.

## Disclosure

All authors have read and approved the final manuscript.

## Ethics Statement

The current research was conducted based on the Declaration of Helsinki (1964) and has been approved by the Ethics Committee of the Research Institute of Physical Education and Sports Sciences (IR.SSRC.REC.1402.244).

## Conflicts of Interest

The authors declare no conflicts of interest.

## Data Availability

The data that support the findings of this study are available from the corresponding author upon reasonable request.
